# Gut Microbiota Composition and Metabolic Potential of Long-Living People in China

**DOI:** 10.3389/fnagi.2022.820108

**Published:** 2022-07-07

**Authors:** Siyuan Zhang, Ruihong Ning, Bo Zeng, Feilong Deng, Fanli Kong, Wei Guo, Jiangchao Zhao, Ying Li

**Affiliations:** ^1^School of Laboratory Medicine/Sichuan Provincial Engineering Laboratory for Prevention and Control Technology of Veterinary Drug Residue in Animal-Origin Food, Chengdu Medical College, Chengdu, China; ^2^Guangdong Provincial Key Laboratory of Animal Molecular Design and Precise Breeding, College of Life Science and Engineering, Foshan University, Foshan, China; ^3^Farm Animal Genetic Resources Exploration and Innovation Key Laboratory of Sichuan Province, Sichuan Agricultural University, Chengdu, China; ^4^College of Life Science, Sichuan Agricultural University, Ya’an, China; ^5^Department of Animal Science, Division of Agriculture, University of Arkansas, Fayetteville, AR, United States

**Keywords:** long-living people, gut microbiota, short chain fatty acid (SCFA), trimethylamine (TMA), butyric acid

## Abstract

Individuals with naturally long-life spans have been extensively studied to gain a greater understanding of what factors contribute to their overall health and ability to delay or avoid certain diseases. Our previous work showed that gut microbiota can be a new avenue in healthy aging studies. In the present study, a total of 86 Chinese individuals were assigned into three groups: the long-living group (90 + years old; *n* = 28), the elderly group (65–75 years old; *n* = 31), and the young group (24–48 years old; *n* = 27). These groups were used to explore the composition and functional genes in the microbiota community by using the metagenomic sequencing method. We found that long-living individuals maintained high diversity in gene composition and functional profiles. Furthermore, their microbiota displays less inter-individual variation than that of elderly adults. In the taxonomic composition, it was shown that long-living people contained more short-chain fatty acid (SCFA)-producing bacteria and a decrease in certain pathogenic bacteria. Functional analysis also showed that the long-living people were enriched in metabolism metabolites methanol, trimethylamine (TMA), and CO_2_ to methane, and lysine biosynthesis, but the genes related to riboflavin (vitamin B2) metabolism and tryptophan biosynthesis were significantly reduced in long-living individuals. Further, we found that long-living people with enriched SCFA- and lactic-producing bacteria and related genes, highly centered on producing key lactic acid genes (*ldhA*) and the genes of lysine that are metabolized to the butyrate pathway. In addition, we compared the gut microbiota signatures of longevity in different regions and found that the composition of the gut microbiota of the long-lived Chinese and Italian people was quite different, but both groups were enriched in genes related to methane production and glucose metabolism. In terms of SCFA metabolism, the Chinese long-living people were enriched with bacteria and genes related to butyric acid production, while the Italian long-living people were enriched with more acetic acid-related genes. These findings suggest that the gut microbiota of Chinese long-living individuals include more SCFA-producing bacteria and genes, metabolizes methanol, TMA, and CO_2_, and contains fewer pathogenic bacteria, thereby potentially contributing to the healthy aging of humans.

## Introduction

Aging is an inevitable process of life, accompanied by a progressive decline in tissue and organ function and an increased risk of mortality (Sen et al., 2016). The aging population poses substantial challenges to society and the health care system (Harper, 2014). With the rapid growth of the elderly population today (He et al., 2016), healthy aging is one way to mitigate sickness in the aging society. In gut microbiome studies, accumulating evidence links host healthy aging or longevity to gut microbiota alterations (Yatsunenko et al., 2012; O’Toole and Jeffery, 2015; Clark and Walker, 2018). Studies on invertebrates, such as *Drosophila melanogaster* and *Caenorhabditis elegans* (Han et al., 2017; Clark and Walker, 2018), and vertebrate animals (*Nothobranchius furzeri*) (Smith et al., 2017) indicate that microbes extend their effects beyond host pathology to systemic modulation of slow aging and prolong life span by originating interspecies signaling (diffusible molecules) or some metabolite molecules. Some studies have demonstrated that the human gastrointestinal tract houses a complex microbial population, and this microbiota is considered a potential determinant of healthy aging in humans and longevity because it can modulate aging-related changes in innate immunity, sarcopenia, and cognitive function, all of which are elements of frailty (O’Toole and Jeffery, 2015; Vaiserman et al., 2017).

Centenarians represent the best model of healthy aging by demonstrating a lower incidence of chronic illness, and a reduction in morbidity (Franceschi and Bonafe, 2003). Both cell culture-dependent (Zhao et al., 2011; Drago et al., 2012) and –independent (Biagi et al., 2016; Kong et al., 2016) studies show that the gut microbiota of long-living people differs from that of the elderly and the young. Previously, our lab study regarding long-living gut microbiota composition by 16S rRNA gene sequencing has shown that long-living people differ from other age groups and, in addition, they possess more beneficial gut bacteria (Kong et al., 2016). Also, studies in Italy and Japan have found that the gut microbiota diversity in long-living individuals is higher than that of young people and found enrichment of some subdominant taxa associated with healthy aging (Biagi et al., 2016; Kong et al., 2019). Shotgun metagenomic analysis methods can infer both taxonomic and functional information from complex microbial communities, guiding phenotypic studies aimed at understanding their potential role in human health and disease (Rampelli et al., 2013; Chen et al., 2021). Wu et al. (2019) and Rampelli et al. (2020) used shotgun metagenomics sequencing to investigate extreme longevity and found a high capacity of glycolysis and related SCFA production and a low abundance of pathways involved in carbohydrate degradation in the gut microbiota which is associated with host health. To understand the profile of the gut microbiome of China’s long-living people and illustrate the function clearly in human healthy aging, we used metagenomics and sequenced fecal samples from 86 subjects, including 28 long-living people (90 + years), 31 elderly people (65–75 years), and 27 young people (24–48 years) that were focused on the gut microbiome composition and function of long-living people in the Dujiangyan (Sichuan Province, China) region with higher rates of longevity.

## Materials and Methods

### Sampling Collection and DNA Extraction

All 86 fecal samples were collected from 86 Chinese individuals living in Dujiangyan, Sichuan Province, China, which have higher rates of longevity in China as indexed in the 6th National Census of China in 2010, including 28 long-living people (group HL, aged 90 + years), 31 elderly people (group HE, aged 65-75 years), and 27 young people (group HY, aged 24–48 years) (Supplementary Table 1). All participants did not take any probiotics (drink yogurt et al.) for 3 months and did not use antibiotic treatments for 6 months. The long-living participants were healthy and independent at enrollment, assessed methods, and other claims were similar to our previous lab study (Kong et al., 2016), and the elderly and young were also healthy. This study was approved by the Ethical Committee of Sichuan Agricultural University (DKY-B20160402). Fecal samples were collected by the participants and refrigerated at home for no more than 1 day before they were taken to Sichuan Agricultural University for storage at −80°C.

A frozen aliquot (200 mg) of each fecal sample was processed, and bacterial DNA was extracted with the QIAamp PowerFecal DNA Kit (Qiagen).

### Metagenomic Sequencing, Assembly, and Gene Catalog Construction

Paired-end metagenomic sequencing was performed on the Illumina NovaSeq 6000 platform with a pair-read length of 150 bp (insert size 350 bp). The sequence reads were first filtered by the in-house pipeline at Annoroad Gene Technology Company. Then, low-quality and human reads (according to alignment to hg19) were removed from the MOCAT2 pipeline (Kultima et al., 2016). The high-quality sequencing reads were then *de novo* assembled into contigs using Megahit v1.2.2 (Li et al., 2016). Open reading frames (ORFs) were identified by Prodigal v.2.6.1 (Hyatt et al., 2010).

Redundant genes were removed using CD-HIT (95% identity, 90% overlap) (Fu et al., 2012). This gene catalog was further compared with an existing gut microbial reference catalog of 11,446,577 genes (IGC) (Xie et al., 2016) using CD-HIT. To reduce biases caused by different sequence depths, high-quality reads of all 86 samples were randomly sub-sampled to 27,481,705 (the minimum number of sequences of the 86 samples) using Seqtk.^1^ Relative abundances of the genes were computed by aligning high-quality sequencing reads to the reference gene catalog as previously described (Qin et al., 2012).

### Species Profile, Functional Annotation, and Construction of Short-Chain Fatty Acids and Lactate Biosynthesis Pathways

The species profiles of the microbiota composition were predicted using mOTU V2 (Milanese et al., 2019) with default parameters.

The non-redundant gene set was translated from nucleotide sequences into amino acid sequences using Biopython. Non-redundant gene sets were annotated against a non-redundant set of the Kyoto Encyclopedia of Genes and Genomes (KEGG) genes database using GhostKOALA (Kanehisa et al., 2016). Abundances of genes that belonged to the same KEGG Orthology (KO) were summed together.

To construct short-chain fatty acids and lactate biosynthesis pathways, we reviewed the literature and those synthesized from Zhao (Zhao et al., 2019), Vital (Vital et al., 2014), and Bourriaud’s (Bourriaud et al., 2005) studies. The final collection of genes was selected by KEGG annotation results.

### Gut Microbiota Signatures of Healthy Aging in Different Regions

To confirm that the replicability and variability of the gut microbiota signatures of the long-living people in Sichaun (China) are consistent in different regions, we downloaded and analyzed the metagenomic data of the long-living people in Sardinia (Italy) (Wu et al., 2019) and Emilia Romagna (Italy) (Rampelli et al., 2020). Wu et al.’s study (2019), included 19 long-living people (group LSI, aged 99 + years), 23 elderly people (group ESI, aged 68-88 years), and 17 young people (group YSI, aged 21–33years) who were living in Sardinia, Italy, and gut microbiome metagenomic sequencing was performed on the Illumina HiSeq X10 PE150 platform (with an average insert size 350 bp), while the sequencing data can be downloaded from PRJEB25514. Rampelli’s study (Rampelli et al., 2020), included 38 long-living people (group LEI, aged 99 + years), 13 elderly people (group EEI, aged 65-75 years), and 11 young people (group YEI, aged 22-48years) who were living in the Emilia Romagna region (Italy), and gut microbiome metagenomic sequencing was performed on the Illumina NextSeq PE150 platform (with an average insert size 350 bp), while sequencing data can be downloaded from PRJNA553191.

The species and function profiles of the gut microbiota composition were predicted using the method above.

### Statistical Analysis

Shannon diversity index and principal-coordinate analysis (PCoA) were performed using R packages ade4 and vegan (version 2.5-5). Shannon index of the gene, species and KOs, gene number, and gene Bray-Curtis were compared between the three age groups using the Mann-Whitney test. ANOVA was performed followed by the Tukey-Kramer multiple-comparison test or Kruskal-Wallis test which was then followed by Dunn’s *post hoc* multiple-comparison test to determine whether significant differences existed in taxa phylum, genus, species, functional pathway, and KOs between the three age groups. LEfSe analyses were used to determine whether significant differences existed between the three age groups. Visualization was done using the R packages VennDiagram, ggplot2, and ggpubr.

## Results

### Gut Microbiome Diversity of the China Long-Live Cohort

**FIGURE 1:**
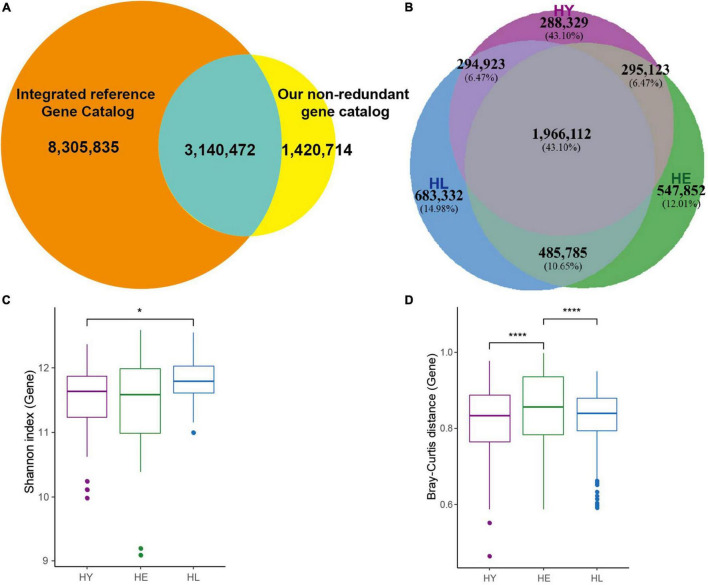
Gut microbiome profile in three age groups. **(A)** Venn diagram of gut microbial genes shared between Integrated reference Gene Catalog (IGC) and our non-redundant genes. **(B)** Venn diagram of gut microbial genes shared between the three age groups. Boxplot of the distribution of gene Shannon diversity index **(C)** and the distribution of inter-individual Bray-Curtis distance **(D)**. Variation between different age groups was detected by Kruskal-Wallis followed by Dunn’s *post hoc* multiple-comparison test. Statistical significance symbols: *, *P* < 0.05; ****, *P* < 0.0001. HL, long-living people; HE, elderly people; HY, young people.

We collected fecal samples from 86 Chinese and divided them into 3 groups according to age (Supplementary Table 1): the long-living group [HL; 95.32 ± 4.22 (SD) years; *n* = 28], elderly group (HE; 70.55 ± 3.13 years; n = 31), and young group (HY; 40.33 ± 7.57 years; *n* = 27). After metagenomic shotgun-sequencing, an average of 65.46 million high-quality non-human reads per sample were obtained, totaling 0.69 terabytes (TB) of sequences (Supplementary Table 1). *De novo* assembly, gene prediction, and gene clustering of these sequences led to a total of 4,561,456 non-redundant genes, ensuring a saturated mapping ratio of the sequencing reads to gene-coding regions (on average 83.81%, Supplementary Table 1). We compared our non-redundant gene sets with IGC, resulting in a catalog of 12,867,301 genes. There were 1,420,714 (31.15%) genes not included in the previous human reference gene catalog (Figure 1A) (Xie et al., 2016). In this, 42.05% (1,918,060 genes) of the genes could be functionally annotated to KEGG orthologous (KOs) groups, similar to previous studies (Li et al., 2014; Xie et al., 2016).

In total, 1,966,112 (43.10%) genes (Figure 1B) were shared among the three groups, while the number of the genes was not different, and the long-living group showed a higher median gene number compared with the young and elderly group (Supplementary Figure 1A). Based on the relative abundance of genes, species, and KOs, we examined the microbial diversities (Shannon indexes) in each age group (Figure 1C and Supplementary Figures 1B,C). Our results indicated that the genes (Figure 1C) and KOs (Supplementary Figure 1B) diversity in the long-living group were significantly higher than the young (*P* < 0.05, Kruskal-Wallis followed by Dunn’s *post hoc* multiple-comparison test), which was not significantly different in the elderly. For species diversity, long-living have a higher median but not significantly compared to the other two groups (Supplementary Figure 1C). However, surprisingly, we discovered that as younger, the gut microbiota of long-living displays less inter-individual variation than that of the elderly adults (Figure 1D).

To investigate the similarity of the gut microbiota for each individual among the three age groups, we used PCoA based on the Bray-Curtis distance of the genes, genus, and KOs relative abundance to visualize the distribution and clustering of the subjects. In our results, these three cluster groups did not clearly separate from each other (Supplementary Figures 1D–F). Analysis of similarities (ANOSIM) test (Supplementary Figures 1D–F) using Bray-Curtis distance revealed that significant difference in the genes and KOs composition of gut microbiota was evident between the long-living and the young (*R* = 0.092, *P* = 0.003; *R* = 0.051, *P* = 0.03) and long-living and the elderly (*R* = 0.043, *P* = 0.05; *R* = 0.035, *P* = 0.053). No significant differences comparing the young and elderly (*R* = −0.016, *P* = 0.77; *R* = −0.02, *P* = 0.86) were observed. Also, there were no significant differences in the genus composition between the three age groups (*P* > 0.05).

### Gut Microbiota Compositional Profiles in the Three Age Groups of China’s Long-Live Cohort

**FIGURE 2:**
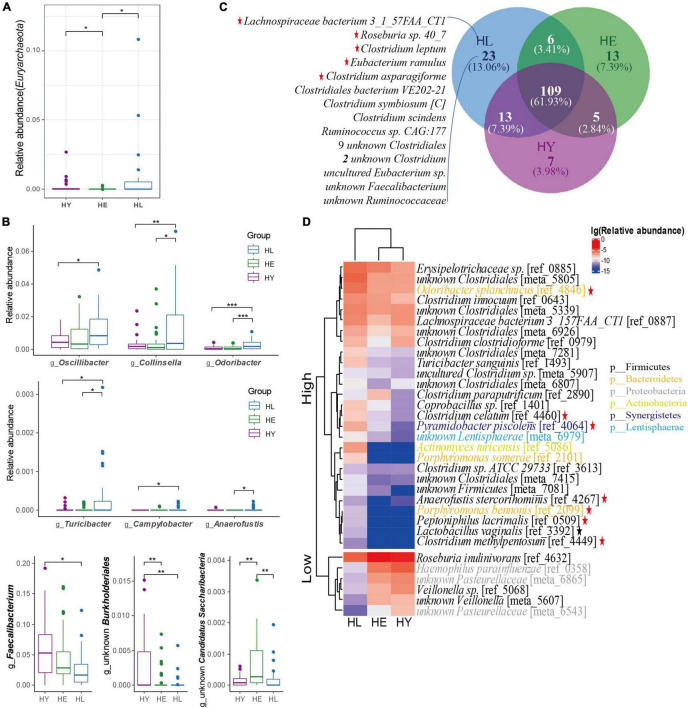
Healthy aging signature of gut microbiota taxonomic components in the three age groups. **(A)** Relative abundance of the *Euryarchaeota* phylum. **(B)** The significantly different genus distributions between the three age groups. **(C)** Core microbiota species distribution in the three age groups. The “Core microbiota” is defined as the species shared by more than 50% of the individuals in each age group. **(D)** Heatmap of the relative abundance of species that are significantly different in the three age groups. The base 10 logarithms of relative abundance were used as input, and complete linkage clustering was used. The distance matrix was created by the “Pearson” method. Kruskal-Wallis followed by Dunn’s *post hoc* multiple-comparison test, *P* < 0.05. Values that are significantly different are indicated by a bar and asterisks as follows: *, *P* < 0.05; **, *P* < 0.01; ***, *P* < 0.001. Red star: SCFA-producing bacteria; Black star: lactic-producing bacteria. Abbreviations: HL, long-living people; HE, elderly people; HY, young people.

We constructed all reads with mOTU V2 (Milanese et al., 2019) to investigate the gut microbiota markers of longevity (Supplementary Table 2). The *Firmicutes*, *Bacteroidetes*, *Actinobacteria*, and *Proteobacteria* bacteria were the dominant phyla in all groups (Supplementary Figure 2). There was a smaller abundance of phyla including *Lentisphaerae*, *Synergistetes*, and *Tenericutes* enriched in the long-living group compared to that of the elderly and young (*P* < 0.05; Supplementary Figure 3A). *Candidatus Saccharibacteria* was enriched in the elderly compared with long-living and young groups. Also, we found that *Euryarchaeota*, an *Archaea* phylum, showed significantly higher abundance in the long-living group and young group (*P* < 0.05; Figure 2A).

At the genus level, from the top 10 genera, 7 genera were similar to Sardinian people (Wu et al., 2019) who were in a high prevalence of longevity, but with subtle differences in relative abundance ordering (Supplementary Figure 4A). We found that *Oscillospira, Collinsella, Odoribacter, Turicibacter, Campylobacter*, and *Anaerofustis*, genera were enriched in the long-living group, while a lower relative abundance of *Faecalibacterium* and an unknown *Burkholderiales* genera were observed in the long-living group compared with that of the elderly and young groups (*P* < 0.05; Figure 2B and Supplementary Table 3). Also, the relative abundance of unknown *Candidatus Saccharibacteria* showed higher abundance in the elderly than in the long-living and young groups (*P* < 001; Figure 2B).

At the species level, we analyzed the “Core microbiota” (defined as microbes that are present in at least 50% of the samples at the species level) to identify the key microorganisms associated with long-living people. Similar to centenarians in Sardinian (Wu et al., 2019), the Chinese long-living group always showed a higher species richness compared with the young and elderly (Figure 2C). Interestingly, 23 core microbiota uniquely seen in the long-living group were mostly related to SCFA-producers (Figure 2C), including butyrate-producing bacterium *Roseburia sp. 40_7*, *Lachnospiraceae bacterium 3_1_57FAA_CT1*, and *Clostridium leptum*; acetate, lactate, and ethanol-producing bacterium *Clostridium asparagiforme*; butyrate and acetate-producing bacterium *Eubacterium ramulus* (Supplementary Table 3).

We then analyzed the relative abundance of species with significant differences between the three different age groups. From all 1,155 species (Supplementary Table 2), compared to the elderly and young groups, we observed a higher abundance of 27 species and a lower abundance of 6 species in the long-living group (ANOVA test, *P* < 0.05; Figure 2D). We noticed that acetic producing bacteria, *Anaerofustis stercorihominis*, *Clostridium celatum*, and *Porphyromonas bennonis*; acetate and propionate producing bacteria, *Odoribacter splanchnicus*, *Clostridium methylpentosum*, and *Pyramidobacter piscolens* were highly abundant in long-living people (Supplementary Table 3). In addition, butyrate-producing bacterial *Odoribacter splanchnicus*, *Peptoniphilus lacrimalis*, and *Anaerofustis stercorihominis* were enriched and *Roseburia inulinivorans* were less enriched in the long-living group (Supplementary Table 3). We also found *Lactobacillus vaginalis* can digest D-galactose, D-glucose, and lactose, producing D/L-lactic acid (Supplementary Table 3). Next, we noticed that long-living people included a low abundance of some pathogenic bacteria, *Haemophilus parainfluenzae* and *Veillonella sp.* (Supplementary Table 3) while containing more SCFA and lactic acid-producing microorganisms compared with the young and elderly. At the same time, long-living people contain fewer opportunistic pathogens *Haemophilus parainfluenzae* and *Veillonella sp.* compared to elderly individuals.

### Potential Metabolic Function of Gut Microbiota Between the Three Age Groups

Previously, it was reported that the functional capacity of the different life stages of gut microbiota contains their own functional preferences. In the gut of long-living people, little is known about microbiota function (Lynch and Pedersen, 2016). In addition, our previous research also found that long-living people are enriched with more SCFA and lactic-producing bacteria. So, we further studied the genetic function of the gut microbiome.

**FIGURE 3:**
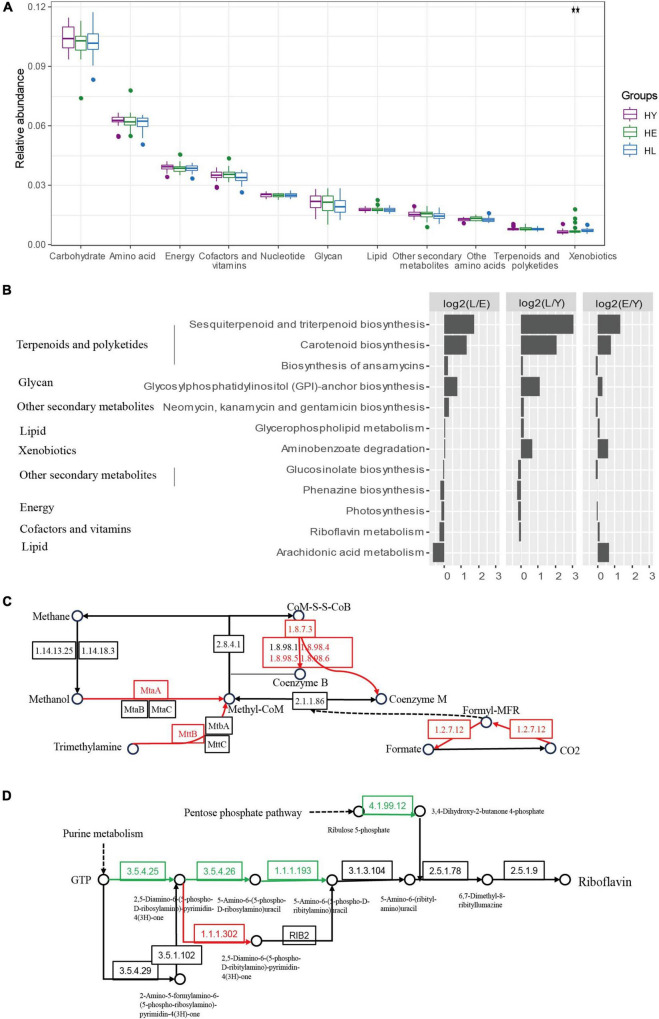
Functional signatures of gut microbiota in the three age groups. **(A)** Relative abundance of kyoto encyclopedia of genes and genomes (KEGG) metabolic pathways of the microbiome in different age groups (ANOVA). **(B)** Relative abundance of the gene pathways that are significantly different in the three age groups (ANOVA, *P* < 0.05). The long-living group compared with the elderly group [HL versus HE (L/E)], the long-living group compared with the young group [HL versus HY (L/Y)], and the elderly group compared with the young group [HE versus HY (E/Y)] are shown in each panel. The length of the bar indicates the base 2 logarithm values of the relative abundance ratios for each age group; 0 represents equal abundance in the two groups. Differences of KEGG orthologous (KOs) in the methane metabolism **(C)** and riboflavin (vitamin B2) metabolism **(D)**. HL, long-living people; HE, elderly people; HY, young people.

We found the most predominant function for gut microbiota metabolism in all three age groups is carbohydrate metabolism, amino acid metabolism, energy metabolism, and metabolism of cofactors and vitamins (Figure 3A). However, we noticed that the relative abundance of the genes related to xenobiotic biodegradation and metabolism increased with age (Figure 3A). KEGG pathways involved in metabolism (Figure 3B; ANOVA test, *P* < 0.05), including sesquiterpenoid and triterpenoid biosynthesis pathways, and the carotenoid biosynthesis, as well as glycosylphosphatidylinositol (GPI)-anchor biosynthesis, were significantly enriched in the long-live group compared to both elderly and young groups.

Further, we analyzed KEGG KOs, 938 of 11,983 KOs were significantly different among the three age groups (ANOVA test, *P* < 0.05), of which 143 KOs showed a significant increase and 10 decreased in the long-living group compared to the elderly and the young age groups (Supplementary Figure 5). KOs enriched by long-living people are concentrated in metabolic pathways related to carbohydrate metabolism, amino acid metabolism, and energy metabolism (Supplementary Figure 5). The enriched KOs specific function was primarily centralized on the metabolism of methanol, CO_2_, and trimethylamine (TMA) to methane (K03388, K03389, K03390, K14080, K11261, and K14083) (Figure 3C).

Additionally, in the gut microbiota of long-living people, we detected a low abundance of riboflavin (vitamin B2) metabolism (Figures 3B,D) and Tryptophan biosynthesis (Supplementary Figure 5), the function of GTP metabolism to riboflavin (K11752, K14652) and tetrahydrofolate (K14652), chorismate to tryptophan (K01657, K01696), and F-type ATPase (K02109) (Figure 3D and Supplementary Figure 5).

### Pathways for Butyrate Biosynthesis in the Gut Microbiome Play an Important Role in Healthy Aging

**FIGURE 4:**
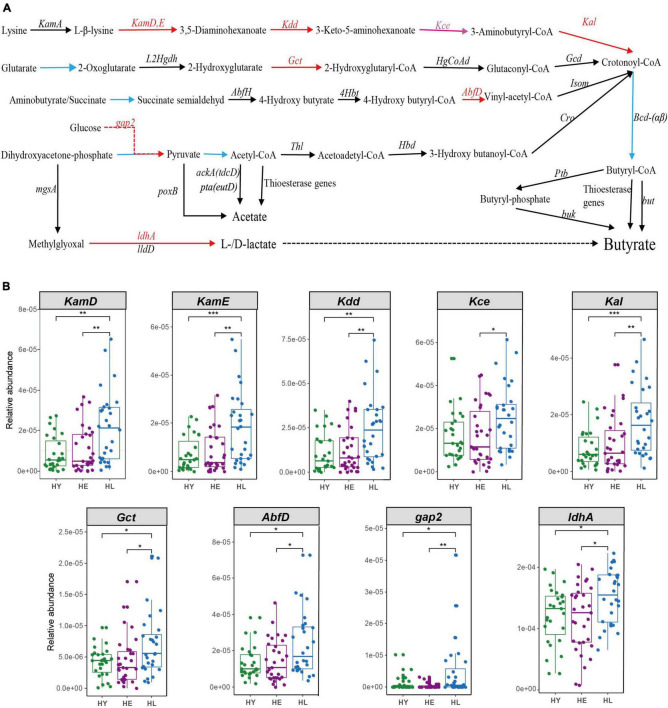
Acetate, butyrate, and lactate biosynthesis pathways **(A)** and the relative abundance of the different genes between the three age groups **(B)**. Pathways for butyrate biosynthesis were drawn according to Vital et al. (2014) and Zhao et al. (2019); Pathways for acetate biosynthesis were drawn according to Zhao et al., 2019); Pathways for lactate biosynthesis were drawn according to Bourriaud et al. (2005). Red arrows, HL > HE HY, *P* < 0.05; pink arrows, HL > HE, *P* < 0.05; black arrows, no significant between the three age groups. The blue line from crotonoyl-CoA to butyryl-CoA was not analyzed, because it was shared by all four pathways leading to Butyryl-CoA. HL, long-living people; HE, elderly people; HY, young people. Statistical significance symbols: *P < 0.05; **P < 0.01; ***P < 0.001.

We found some enriched KOs including lysine biosynthesis, lysine degradation and metabolism, that act on lysine (K00928, K05822) and pyruvate (K00150) production, which are the principal metabolic material for butyrate production. Gut microbiota composition also found long-living enrich more SCFA- and lactic-producing bacteria (Such as *O. splanchnicus et al*.). Thus, we constructed pathways for short-chain fatty acids and lactate biosynthesis (Bourriaud et al., 2005; Vital et al., 2014; Zhao et al., 2019) (Figure 4 and Supplementary Table 4). We found that long-living people show enrichment in key genes for all pathways producing butyrate, especially the lysine to butyrate pathway (*KamD, KamE, Kdd*, and *Kal*; *Kce* were significantly different between the long-lived and elderly) (Figure 4). Simultaneously, we found that NAD + -dependent lactate dehydrogenase (LDH, encoded by *ldhA*), the major contributor to lactate production (Zhao et al., 2019), was significantly enriched in the long-living group compared to the elderly and young (Figure 4).

### The Replicability and Variability of the Gut Microbiota Signatures in the Long-Living People

**FIGURE 5:**
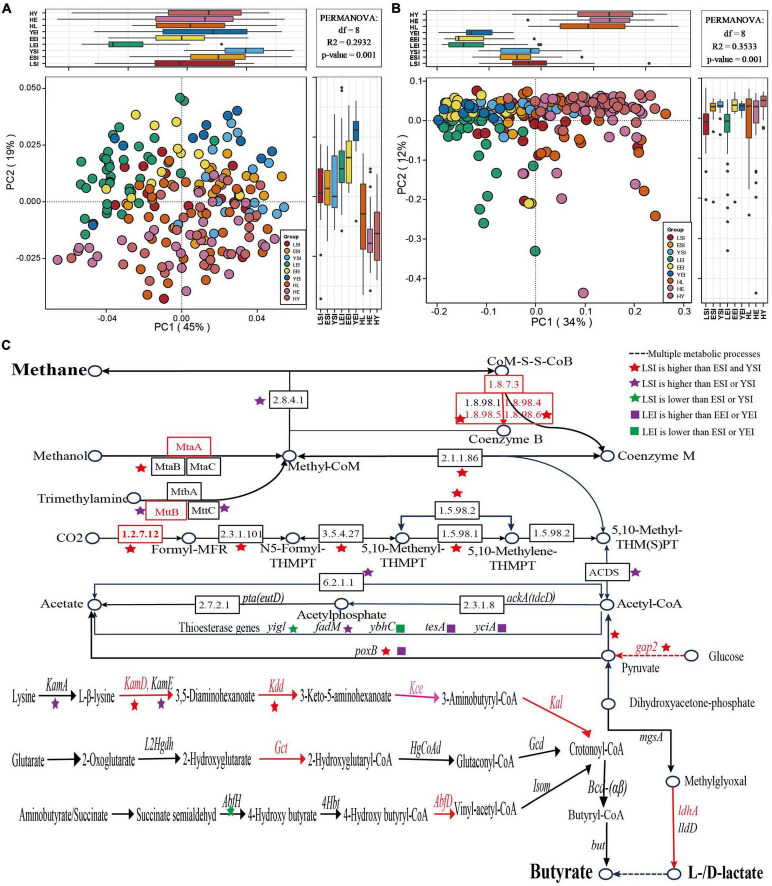
Gut microbiota signatures of healthy aging in different regions. **(A,B)** principal-coordinate analysis (PCoA) based on the Bray-Curtis distance derived from the relative abundance of the species **(A)** and KEGG orthologous (KOs) **(B)** were plotted for the gut microbiota composition and function at the species and KOs level. **(C)** Differences of KOs in the methanogenesis and acetate, butyrate, and lactate biosynthesis pathways. HL, Chinese long-living group; HE, Chinese elderly group; HY, Chinese young group; LSI, Sardinia (Italian) long-living group; ESI, Sardinia (Italian) elderly group; YSI, Sardinia (Italian) young group; LEI, Emilia Romagna (Italian) elderly group; EEI, Emilia Romagna (Italian); YEI, Emilia Romagna (Italian) young group.

First, we focus on the gut microbiome composition (Supplementary Table 2) and functional profile, and we found that Sichuan (China), Sardinia (Italy), and Emilia Romagna (Italy) can clearly separate from each other (Figures 5A,B), while regions (species, *R*^2^ = 0.08065, *P* = 0.001; KOs, *R*^2^ = 0.31203, *P* = 0.001) and age (*R*^2^ = 0.02600, *P* = 0.001; *R*^2^ = 0.02377, *P* = 0.001) levels were among the strongest explanatory factors compared to gender (*R*^2^ = 0.00525, *P* = 0.157; *R*^2^ = 0.00533, *P* = 0.11).

Then, we focus on the taxonomic signatures’ compositions of the gut microbiome in different regions. We found that the phyla (Supplementary Figure 3) and genera (Supplementary Figures 4, 6) of the gut microbiome in the three regions were relatively similar, but differed greatly at the species level (Figure 2D and Supplementary Figure 7). Mainly, the phyla *Euryarchaeota* and *Synergistetes* were enriched in the long-living people in the three studies, but the relative abundance of the dominant phyla (*Firmicutes*, *Bacteroidetes*) of the gut microbiome decreased in both regions of Italy (Supplementary Figure 3). For the top 10 genera with the highest relative abundance, we found that 5 genera were consistent in the three regions, and even 9 genera in Sichuan (China) were the same as those in Sardinia (Italy) (Supplementary Figures 4A,B), while only 6 genera were consistent in the two Italian regions (Supplementary Figures 4B,C). The genus-level statistical test between the three age groups in each of the three regions showed that the content of *Faecalibacterium* in the long-living people was fewer in all regions, while the abundance of butyrate-related genus *Oscillibacter* in Sardinia was low (Figure 2B and Supplementary Figure 6). In addition, we also found that both the long-living people in two Italian regions showed enrichment of *Methanobre*, but reduced *Butyricicoccus*, *Lachnospiroecae*, and *Roseburia* (Supplementary Figure 6). At the species level, in Sardinia (Italy), 18 bacterial species were enriched in long-lived elderly people, and 16 species were relatively low in abundance (ANOVA test, *P* < 0.05; Supplementary Figure 7A). Emilia Romagna (Italy), 44 strains of the gut microbiome in the long-living people were significantly lower than those of the elderly and young (ANOVA test, *P* < 0.05; Supplementary Figure 7B). No common characteristics of gut microbiota were found in the three regions, and the gut microbiota of the long-living people in the two different regions (Sardinia and Emilia Romagna) of Italy did not show enrichment of lactic acid and butyric acid-related bacteria (Figure 2D and Supplementary Figure 7).

Further, we focused on the function of the signatures of the gut microbiome of long-living people in three regions. We analyzed KEGG KOs and found that 1,590 of 13,608 KOs were significantly different among the three age groups (ANOVA test, *P* < 0.05), of which 477 KOs showed a significant increase and 173 decreased in the long-living group compared to the elderly and young age groups in the Sardinia (Italy) regions, and 857 of 11,694 KOs were significantly different among the three age groups (ANOVA test, *P* < 0.05), of which 30 KOs showed a significant increase and 32 decreased in the long-living group compared to the elderly and young age groups in Emilia Romagna (Italy) regions. In Sardinia, the metabolic function of the gut microbiome of the long-living people is more focused toward concentrated in the methane metabolism, using CO2, acetate, methanol, and trimethylamine to generate methane (K00200, K00202, K00203, K00205, K00319, K00577, K00579, K00580, K00581, K00584, K00672, K01499, K11260, K13942, K14128, K22516, and K04480), and also some glycolysis and energy metabolism, including pyruvate (K00150, K15635, and K16306) and lysine (K05828, K05831, K00133, and K00133) biosynthesis et al., which is consistent with Wu’s previous analysis results Wu et al. (2019). In Emilia Romagna (Italy), the metabolic function of the gut microbiome of the long-living people is more concentrated in the glycolysis of sugar to produce pyruvate (K00150) to acetyl-CoA (K00382, K00627, and K00382), etc. (K00963, K00963, K00963, K00150, and K00382) and contains less functional genes for H_2_S production (K00955 and K00957).

Finally, we compared the enrichment of lactic acid and SCFA production-related genes in long-living people in different regions. We found that the Italian long-living people are not enriched in genes related to the production of lactic acid and butyric acid as the Chinese long-living (Figures 4, 5C), while the Italian long-living people are more enriched in acetogenic-related genes and partially in butyric-related genes (Figure 5C). Sardinian long-living people are more enriched in genes related to ‘sugar-pyruvate-acetic acid’ (*gap2*, *poxB*), and lysine metabolism (*KamD*, *Kdd*) than other age groups, while acetyl-CoA to acetate (*fadM*) and lysine metabolism (*KamA*) -related genes are enriched in elderly (Figure 5C). In addition, two genes related to the production of acetate (*yigl*) and butyrate (*AbfH*) were lower than those of the elderly or young (Figure 5C). In Emilia Romagna (Italy), the long-living people were more enriched in pyruvate and acetyl-CoA-to-acetate-related genes (*poxB*, *tesA*, *yciA*) than young adults (Figure 5C).

## Discussion

Our work focused on the gut microbiome composition and function of long-living people (as the best model of “successful aging”) in China (Dujiangyan, Sichuan Province) region with higher rates of longevity (Kong et al., 2016) to better understand the relationship between the gut microbiome and host healthy aging. We found that 31.15% of genes in the gut microbiome of people living in the region with higher rates of longevity were not included in the previous human gut microbiome gene catalog (Xie et al., 2016).

Microbial diversity has been used as a measure of a healthy microbiome (Lloyd-Price et al., 2016). The diversity of the gut microbiome in long-living people was greater than that in young adults which had been verified in Chinese, Italian, and Japanese using 16S rRNA technology (Kong et al., 2019). Comparing the three age groups in this study, we found the genes and KOs Shannon index in long-living individuals was greater than in the young group (Figure 1C and Supplementary Figure 1C), but no significant difference in species diversity by the Shannon index was observed (Supplementary Figure 1B). Also, the study of the Sardinian centenarians using metagenomics sequencing was similar to ours (Wu et al., 2019). This may be affected by the method of species identification, even though mOTUs2 can profile >7,700 microbial species, there are more than 50% of gut microbial species lack representative reference genomes which can restrict the profiling of the composition of microbial communities from metagenomic shotgun sequencing data (Milanese et al., 2019).

For the taxa and function profile, there were some differences among the three age groups, but they could not be clearly separated (Supplementary Figures 1D–F). In our previous study, we found that the gut microbiota in healthy long-living people was significantly isolated from unhealthy individuals (Zhang et al., 2021). This result supports the point of view that dysbiotic individuals vary more in microbial community composition than healthy individuals (Zaneveld et al., 2017). But, unlike Wu’s findings, the young and elderly Sardinians shared similar gut microbiota taxonomic and functional profiles that were different from centenarians (Wu et al., 2019). An interesting result is that we found the gut microbiota of long-living people displays less inter-individual variation than that of elderly adults (Figure 1D). Studies regarding older people (> 65 years) show that the composition of their gut microbiota was greater in inter-individual variation compared with that of younger adults (Claesson et al., 2011). Another big data analysis supports the theory that starting from middle age and old age, the gut microbiome becomes progressively unique over time (Wilmanski et al., 2021). Long-living people showed less inter-individual variation, this may be because the long-living people have a more similar lifestyle and health status.

Furthermore, we found that *Euryarchaeota*, an *Archaea* phylum, showed significantly higher abundance in the long-living and young groups than in the elderly (Figure 2A). At the same time, we also found enriched *Euryarchaeota* in long-living people in different regions of Italy (Supplementary Figures 3B,C). Up to 10% of anaerobic microorganisms in the human gut are methanogenic archaea (Moissl-Eichinger et al., 2018) and are potentially beneficial or harmful to human health (Chaudhary et al., 2018). Some research found that the diversity and abundance of archaea in the human gut were positively correlated with age, and the interaction of archaea with the host and symbiotic bacteria can affect the physiological health of the host [such as consumption of trimethylamine (TMA)], and no pathogenic archaea have been found (Borrel et al., 2020). Incidentally, in gut microbiome function, we found that long-living people were enriched in the methane metabolism pathway, and metabolism of methanol, TMA, and CO_2_ to produce methane (Figure 3C and Supplementary Figure 5). Some studies prove that the *Archaea* in the human gut microbiome can utilize TMA (Borrel et al., 2017). The gut microbiome can convert dietary choline and other substances into TMA, which is then metabolized by the host liver to trimethylamine oxide (TMAO), linked with an increased risk of cardiovascular disease (CVD) (Abbasi, 2019). The prevalence of *Methanomassiliicoccales*, which uses H_2_ to reduce TMA in methanogenesis, was found to correlate positively with the number of different TMA- producing pathways (Borrel et al., 2017). We hypothesize that healthy aging, long-living people with reduced risk of CVD may have more *Archaea* in the methane metabolism pathway to help reduce TMA.

Moreover, we found that long-living people are also enriched in the metabolism of terpenoids and polyketides, including sesquiterpenoid and triterpenoid biosynthesis pathways and carotenoid biosynthesis. Phytochemicals such as alkaloids, polyphenols, and terpenoids found in plants as caloric restriction, fasting, and exercise can improve health and longevity by protecting cells and organs against damage, mutations, and reactive oxygen species (Martel et al., 2019). Higher intake of retinol and some carotenoids were also associated with a reduction in cutaneous squamous cell carcinoma (SCC) risk (Kim et al., 2019). These results indicate that the sesquiterpenoid and triterpenoid biosynthesis pathways and the carotenoid biosynthesis pathway may be important for human health in long-living people.

Besides, we found that the long-living group has a low abundance of the riboflavin (vitamin B2) metabolism pathway (Figures 3B,D). Comparatively, Wu’s study found that the riboflavin synthesis pathway was highly enriched in the centenarians (Wu et al., 2019). As we know, vitamin B2 has an influence on skeletal muscle function (Ticinesi et al., 2017) and is beneficial to host health (Powers, 2003). Also, a study on the human gut microbiome shows that the western diet was related to vitamin biosynthesis (Lynch and Pedersen, 2016). Given the difference in the relative abundance of genes encoding vitamin B2 between Chinese and Italian long-living individuals, and considering the bypass pathway of B2 synthesis is enriched by the enzyme (K14654) (Figure 3D), we assume that the differences may be due to the differences between the Chinese and western diet, or vitamin B2 may be synthesized through the bypass pathway by enzyme K14654.

Previously (Kong et al., 2016; Wu et al., 2019) and in our study, we found that long-living people contain more SCFA-producing bacteria. SCFAs are the main organic acids present in the intestines and were proven to be beneficial for human health (Yang et al., 2020). SCFA-producing bacteria as beneficial commensal microbes may be related to aging (Biragyn and Ferrucci, 2018). Aon *et al.’*s experiment show that SCFA (propionic acid and butyric acid) metabolism is a key pathway for caloric restriction (CR) that can enhance health and lifespan (Aon et al., 2020). The use of metagenomics to study extreme longevity in Italians also revealed that the microbiota in centenarians had a high capacity for glycolysis and fermentation of SCFAs (Wu et al., 2019). It is important that we found long-living people enriched in SCFA-producing taxa, such as *Oscillospira, Odoribacter, Anaerofustis* genera, and species of *Clostridium celatum, Clostridium methylpentosum, Pyramidobacter piscolens, Peptoniphilus lacrimalis, Odoribacter splanchnicus* and *A. stercorihominis* (Figures 2B,D and Supplementary Table 3). Especially, the “Core microbiome” in long-living people also was SCFA-producing taxa (Figure 2C and Supplementary Table 3). In our lab, previous studies showed that long-living people were enriched with SCFA-producing bacteria (*Clostridium* cluster XIVa) (Kong et al., 2016). In this study, we found species *C. celatum* and *C. methylpentosum* were enriched in long-living people. *Odoribacter* which can produce SCFA by fermenting carbohydrates *in vitro* was associated with blood pressure and metabolism (Gomez-Arango et al., 2016), and researchers found similar enrichment in Italian long-living individuals (Biagi et al., 2017). The *Faecalibacterium* genus was low in abundance in long-living people no matter the geographical location or use of different research methods (Biagi et al., 2016; Wu et al., 2019). This result indicates that SCFA-producing bacteria could be a candidate to use as a probiotic for human healthy aging.

In our study, we found that the long-living people’s gut microbiota included more key genes for producing butyrate, especially the lysine to Crotonoyl-CoA pathway (Figure 4). Butyric acid, an SCFA, is the final metabolite in the mammalian gut by fermentation of dietary fiber. It is a key mediator of host-microbe crosstalk in host energy metabolism and immune functions and also in host brain function and behavior (Stilling et al., 2016). Fachi et al. (2019) interventions and restoration of butyrate in infected mice show that butyrate intestinal levels mitigate clinical and pathological features of *Clostridium* difficile-induced colitis through a HIF-1-dependent mechanism. Oral butyrate acts on the intestinal-brain neural circuit to improve energy metabolism by reducing energy intake and enhancing fat oxidation by activating brown adipose tissue (BAT), thereby preventing diet-induced obesity, hyperinsulinemia, hypertriglyceridemia, and hepatic steatosis (Li et al., 2018). Using sodium butyrate to treat germ-free mice found that the sodium butyrate can increase BDNF expression, the abundance of FGF21, and the number of DCX^+^ neurons, also activate the AMPK pathway and produce high expression of SIRT1 in the liver, all of which may relate to host longevity (Kundu et al., 2019). Decreases in the butyric acid-producing bacteria in the gut may lead to senile weakness, and induce Alzheimer’s disease, Parkinson’s disease, and other senile diseases (Haran and McCormick, 2021). Our research speculates that the gut microbiota of long-living people was enriched with more butyrate-producing bacteria and genes, which is beneficial to reducing the occurrence of neurodegenerative diseases and maintaining host health.

Simultaneously, we found NAD + -dependent lactate dehydrogenase (LDH, encoded by *ldhA*), the major contributor to lactate production (Zhao et al., 2019), was significantly enriched in the long-living individuals compared to the elderly and young (Figure 4). Lactate is a very common metabolite with complex functions in the human body. Lactate can be converted to butyrate by gut microbiota and rat lactate has a protective effect on intestinal ischemia, similar to the effect of butyrate (Bertacco et al., 2017). In the above taxa profile, we also found D-/L-lactic-producing bacterial *Lactobacillus vaginalis* enriched in long-living people. These results suggest that butyric-producing bacteria or butyric acid play an important role in the healthy aging of long-living people.

In addition, we found that the taxa and functional composition of the gut microbiota vary greatly in different regions (Figures 5A,B). Previous studies examining the gut microbiota of healthy people from different countries and different sick people in the same province have found that geography is one of the largest influencing factors (He et al., 2018). Two comprehensive studies analyzed the relationship between gut microbiota and host healthy aging and found that geography was the most important factor affecting gut microbiota (Ghosh et al., 2020; Chen et al., 2022). Therefore, the composition of the gut microbiome in different countries or regions is quite different. Interestingly, we found that the gut microbiota of long-living people in different regions also had the same characteristics. Bacteria and genes related to methanogenesis and glucose metabolism are enriched in long-living people in Sichuan, China, and Sardinia and Elman in Italy. Just as previous studies have found that the gut microbiota is in line with the “Anna Karenina principle,” the same healthy people have the same health-related characteristics (Zaneveld et al., 2017). Importantly, we found that the gut microbiome of Chinese long-living people was more enriched in genes related to lactate and butyrate production, while Italian long-lived were more enriched in genes related to acetate production. This is also consistent with our previous research finding that the Chinese long-lived are enriched with more bacteria that are beneficial to the host (Figures 2B–D). The long-living people in China and Italy are enriched in bacteria and genes related to methane production, but bacteria and genes related to lactic acid production and short-chain fatty have different characteristics in different regions, which can provide a reference for predicting healthy aging in different regions.

In summary, our work focused on the composition and function of the gut microbiome in long-living people compared to elderly and young people. We found that the gut microbiome of long-living people differed from the elderly, with higher diversity in gene composition and functional profiles, and displays less inter-individual variation than that of elderly adults. The gut microbiome in long-living people is enriched in *Euryarchaeota* and genes that can metabolize methanol, CO2, and TMA, and reduce the content of some pathogenic bacteria (*Haemophilus parainfluenzae* and *Veillonella sp.*). Most importantly, we found the gut microbiome of long-living people enriches greater SCFA- and lactic-producing bacteria and related genes (*ldhA*, *KamD, KamE, Kidd*, and *Kal*) than that of elderly and younger adults. Therefore, we believe that the gut microbiome in long-living people promotes human health and longevity by enriching more probiotics, and metabolites and reducing the abundance of opportunistic pathogens.

## Data Availability Statement

The datasets presented in this study can be found in online repositories. The names of the repository/repositories and accession number(s) can be found in the article/Supplementary Material.

## Ethics Statement

The studies involving human participants were reviewed and approved by Ethical Committee of Sichuan Agricultural University. The patients/participants provided their written informed consent to participate in this study.

## Author Contributions

YL and SZ conceived and designed the experiments. SZ, RN, BZ, and FD were involved in the data analysis. SZ, RN, BZ, FK, and WG were responsible for statistical analyses. SZ and RN wrote the first draft of the manuscript. JZ and YL wrote sections of the manuscript. All authors contributed to the article and approved the submitted version.

## Conflict of Interest

The authors declare that the research was conducted in the absence of any commercial or financial relationships that could be construed as a potential conflict of interest.

## Publisher’s Note

All claims expressed in this article are solely those of the authors and do not necessarily represent those of their affiliated organizations, or those of the publisher, the editors and the reviewers. Any product that may be evaluated in this article, or claim that may be made by its manufacturer, is not guaranteed or endorsed by the publisher.

## Footnotes

1
https://github.com/lh3/seqtk
