# Supplementary material for: Gut Microbiota Composition and Metabolic Potential of Long-Living People in China
Source: Front Aging Neurosci. 2022 Jul 7;14:820108. doi: 10.3389/fnagi.2022.820108 (PMC9300991; doi:10.3389/fnagi.2022.820108)
Supplement: Supplementary file 1 [file Image_1.pdf]

Supplemental Material

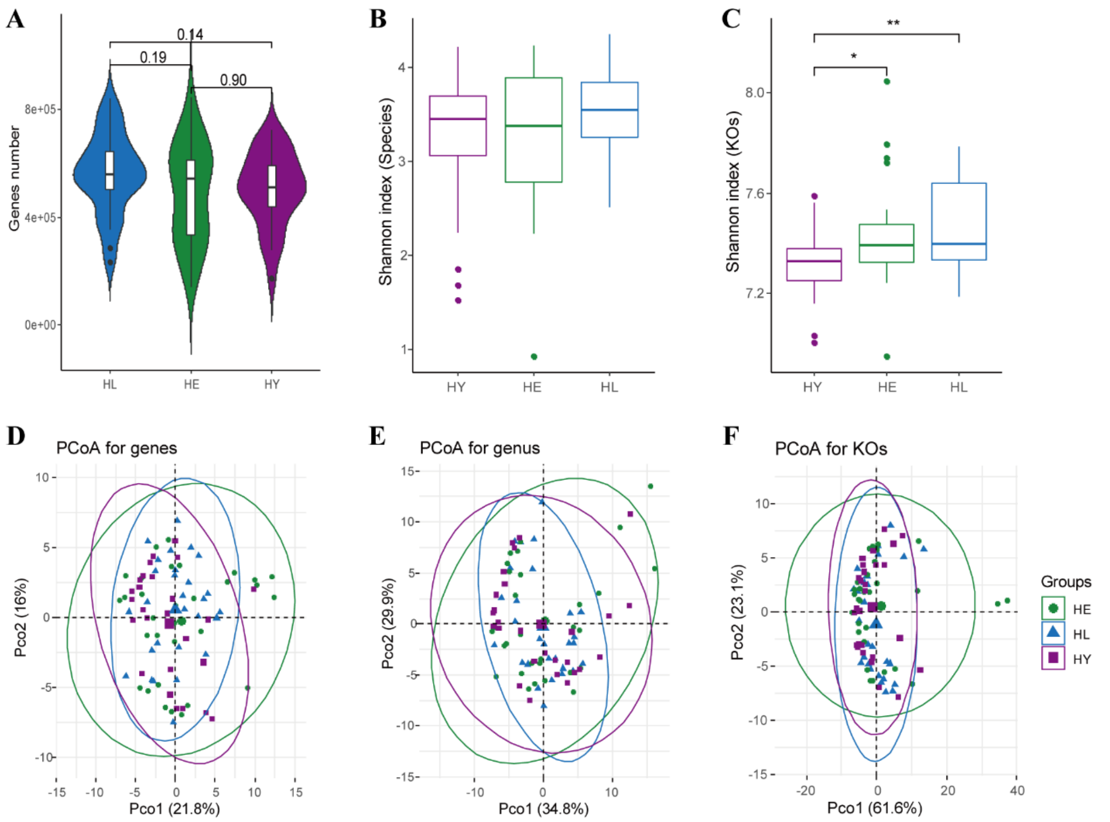

Figure S1. gut microbiota components and functional profiles in the three age groups. (A): Violin Plot of the distribution of the gene number between the three age groups. **(B and C):** Boxplot of the distribution of the species Shannon diversity index (B) and of the distribution of KOs Shannon diversity index (C). Variation between different age groups was detected by Kruskal-Wallis followed by Dunn's post-hoc multiple-comparison test. Statistical significance symbols: *, $P < 0.05$; **, $P < 0.01$; ***, $P < 0.001$. **(D - F):** PCoA based on the Bray-Curtis distance derived from the relative abundance of the genes, genus and KOs were plotted for the gut microbiota composition and functional at the genes, genus and KOs level. Ellipses with 95% confidence around the centroid of each age group are plotted in PCoA.

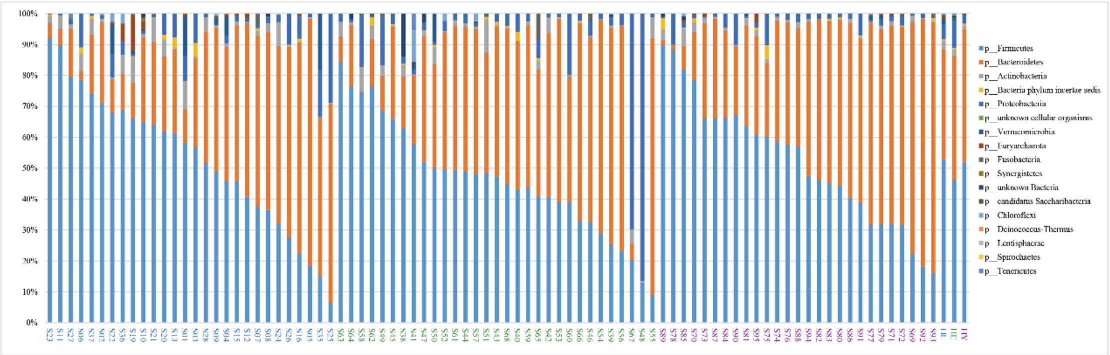

Figure S2. Bacterial taxa composition in the phylum level. Relative abundance of the phylum level gut bacterial taxa per sample, the first 28 were HL individuals, then

31 were HE individuals, then 31 were HY individuals, last three were the average of HL, HE and HY groups.

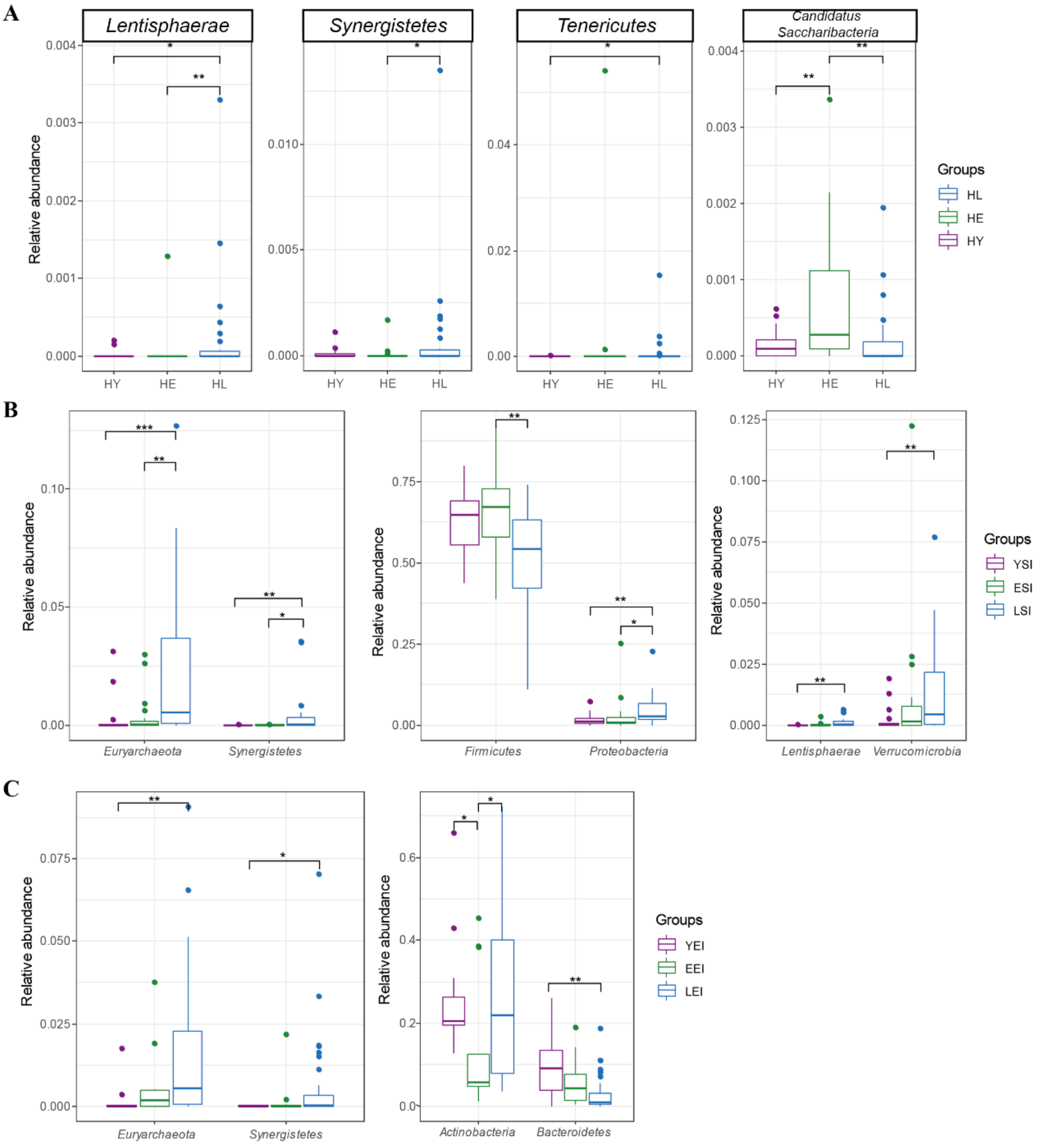

Figure S3. Healthy aging signature of gut microbiota at the phylum level between the three age groups in three regions**. (A):** The difference in the relative abundance of *Lentisphaerae*, *Tenericutes*, *Synergistetes* and *Candidatus Saccharibacteria* phylum at Sichuan (China). **(B):** The difference in the relative abundance of *Lentisphaerae*, *Tenericutes*, *Synergistetes* and *Candidatus Saccharibacteria* phylum at Sardinia (Italy). **(C):** The difference in the relative abundance of *Lentisphaerae*, *Tenericutes*, *Synergistetes* and *Candidatus Saccharibacteria* phylum at Emilia Romagna (Italy). Abbreviations: HL, Chinese long-living group; HE, Chinese elderly group; HY, Chinese young group; LSI, Sardinia (Italian) long-living group; ESI, Sardinia (Italian) elderly group; YSI, Sardinia (Italian) young group; LEI, Emilia Romagna (Italian) elderly group; EEI, Emilia Romagna (Italian); YEI, Emilia

Romagna (Italian) young group.

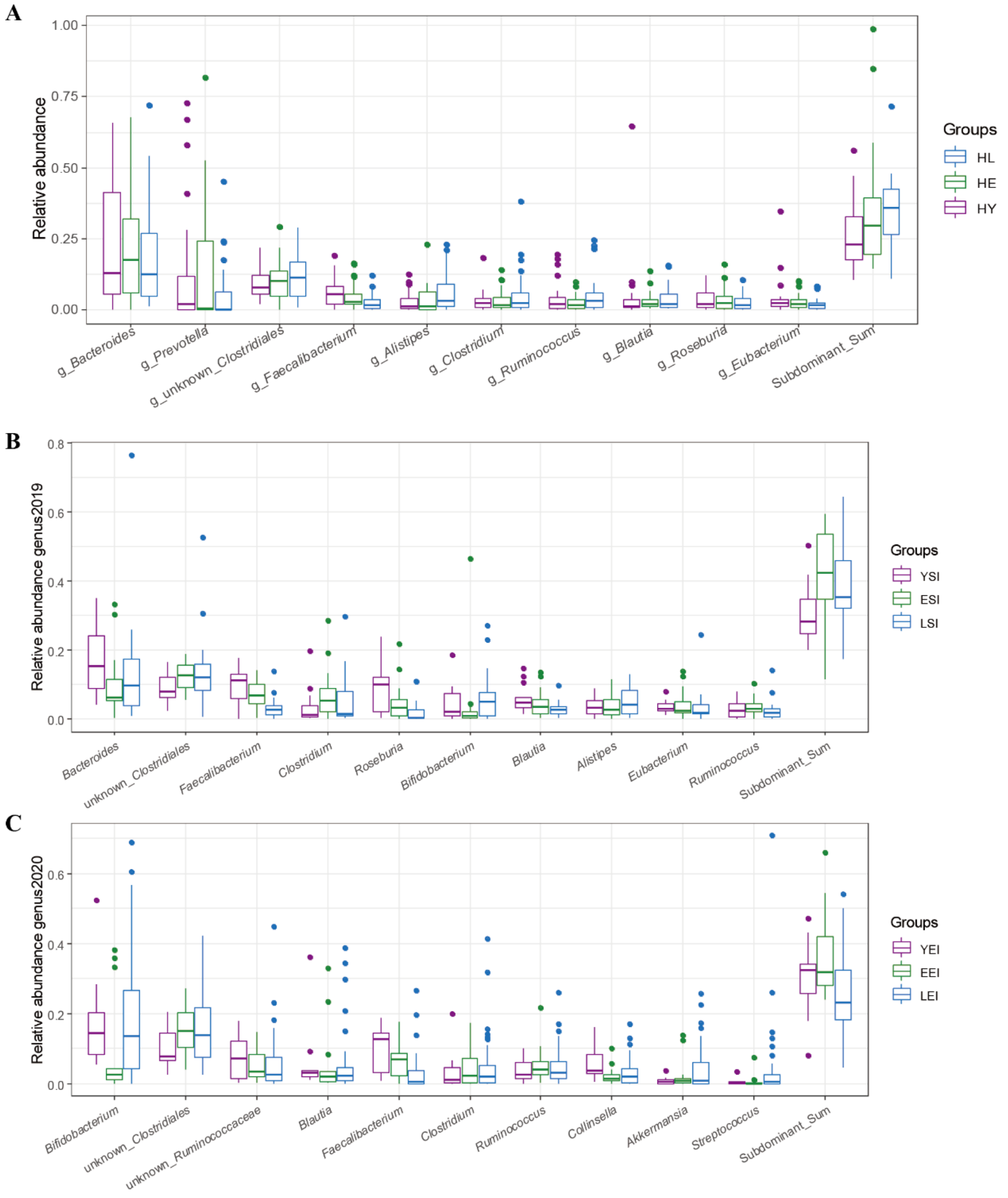

Figure S4. Relative abundance of the top 10 genera in the three age groups at Sichuan (China, A), Sardinia (Italy, B) and Emilia Romagna (Italy, C). Other low-abundant genera are summed into one group labeled “Subdominant_Sum.” Abbreviations: HL, Chinese long-living group; HE, Chinese elderly group; HY, Chinese young group; LSI, Sardinia (Italian) long-living group; ESI, Sardinia (Italian) elderly group; YSI, Sardinia (Italian) young group; LEI, Emilia Romagna (Italian) elderly group; EEI, Emilia Romagna (Italian); YEI, Emilia Romagna (Italian) young group.

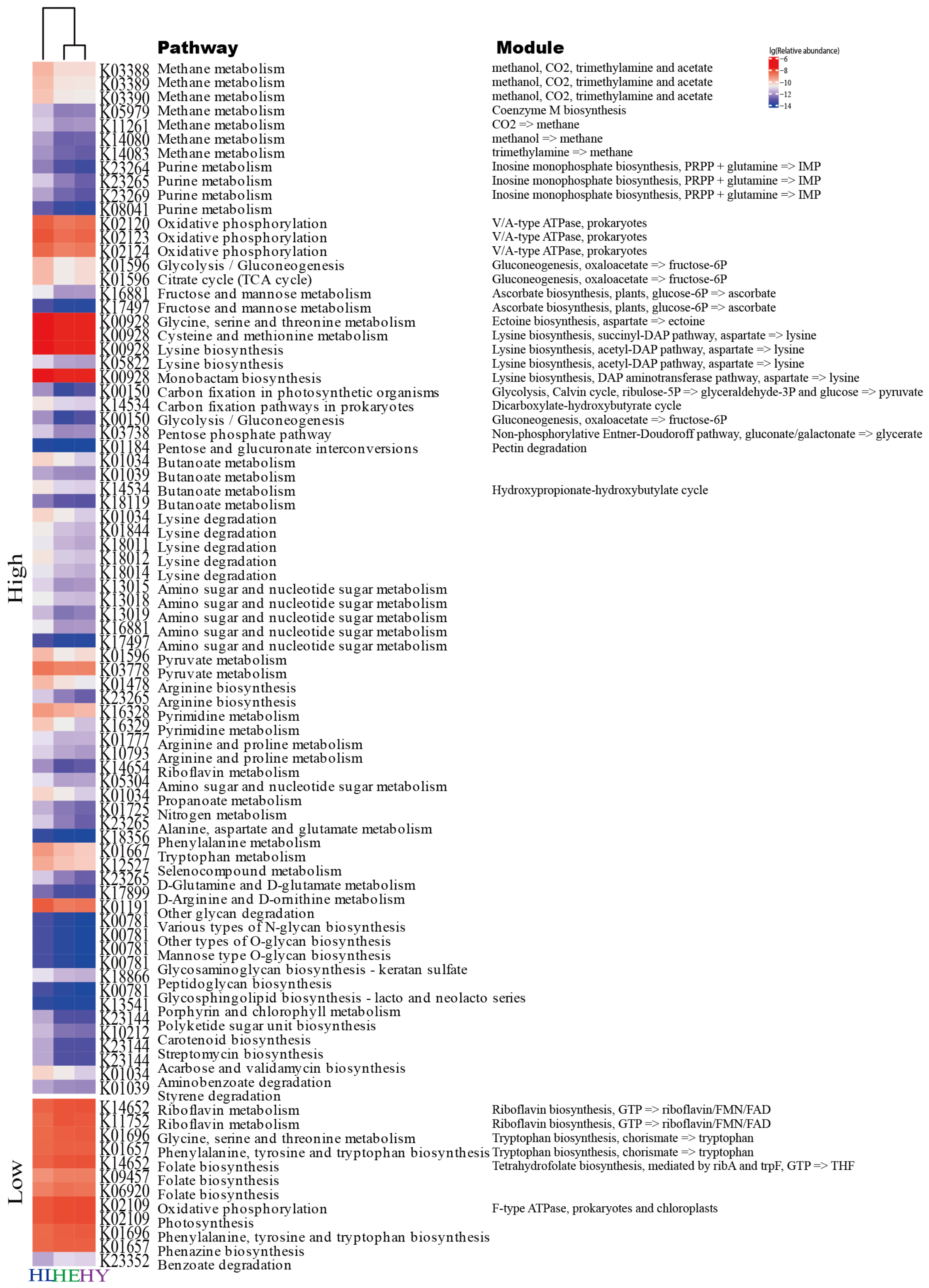

Figure S5. Heatmap of the relative abundance of KOs that are significantly different in the three age groups. The base 10 logarithm of relative abundance was used as input, and complete linkage clustering was used. The distance matrix was created by the "Pearson" method.

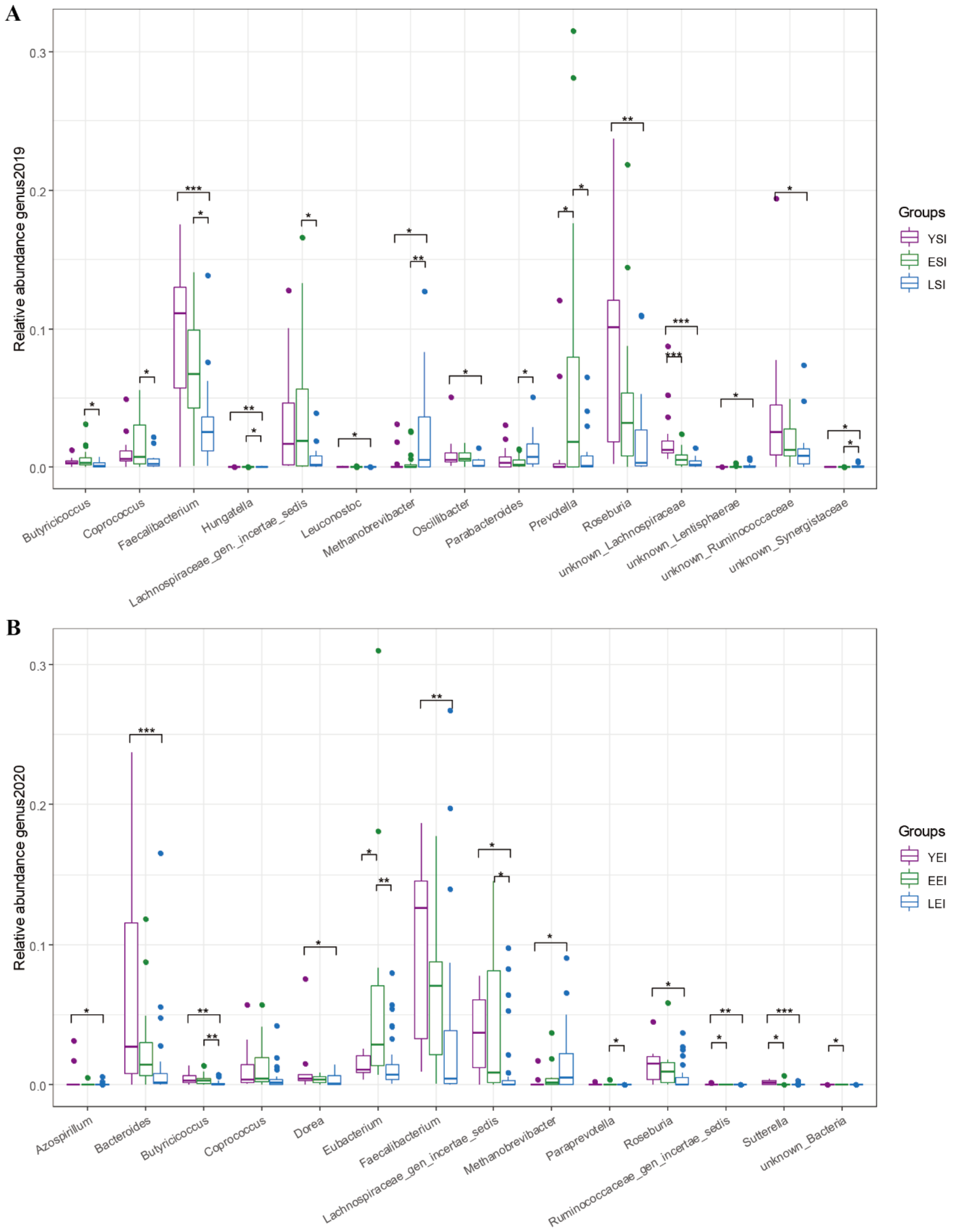

Figure S6. The significantly different genus distributions between the three age groups at Sardinia (Italy, A) and Emilia Romagna (Italy, B). The genera with significantly different distributions among the three age groups detected by the ANOVA test (P<0.05) are marked with an asterisk. Abbreviations: LSI, Sardinia (Italian) long-living group; ESI, Sardinia (Italian) elderly group; YSI, Sardinia (Italian) young group; LEI, Emilia Romagna (Italian) elderly group; EEI, Emilia Romagna (Italian); YEI, Emilia Romagna (Italian) young group.

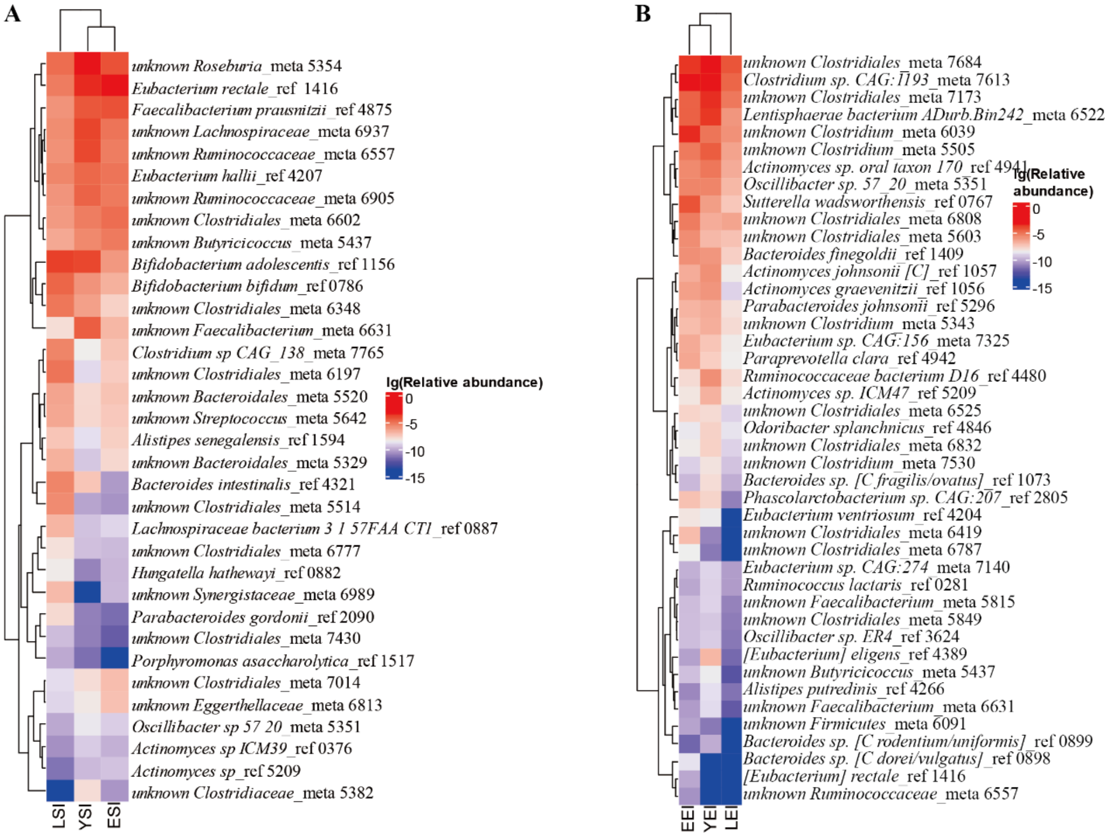

Figure S7. Heatmap of the relative abundance of species that are significantly different in the three age groups at Sardinia (Italy, A) and Emilia Romagna (Italy, B). The base 10 logarithm of relative abundance was used as input, and complete linkage clustering was used. The distance matrix was created by the "Pearson" method. Abbreviations: LSI, Sardinia (Italian) long-living group; ESI, Sardinia (Italian) elderly group; YSI, Sardinia (Italian) young group; LEI, Emilia Romagna (Italian) elderly group; EEI, Emilia Romagna (Italian); YEI, Emilia Romagna (Italian) young group.

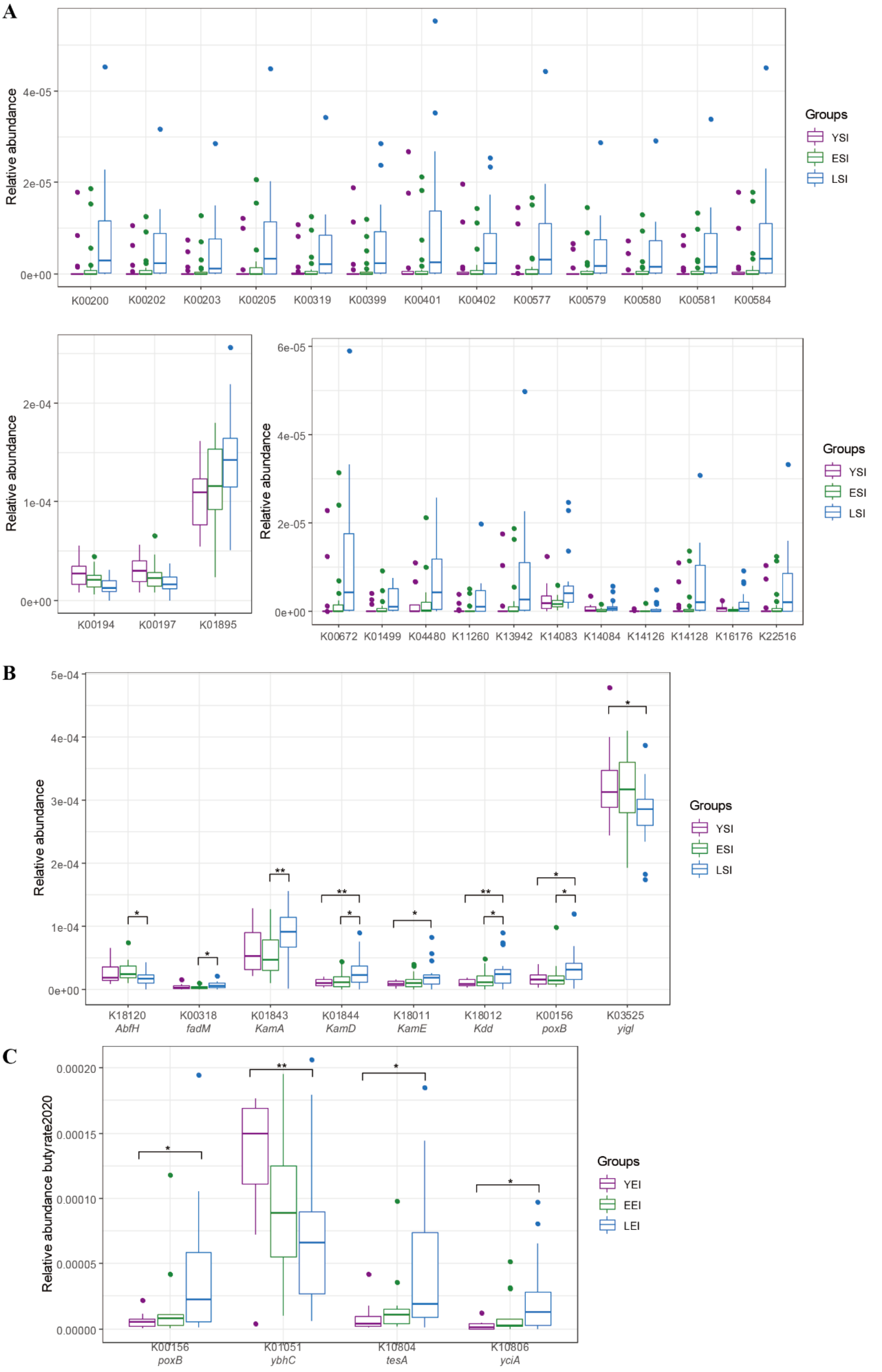

Figure S8. Differences of KOs of the methanogenesis (A) and acetate, butyrate and lactate biosynthesis (B and C) pathways in Sardinia (Italy) and Emilia Romagna

(Italy). Abbreviations: LSI, Sardinia (Italian) long-living group; ESI, Sardinia (Italian) elderly group; YSI, Sardinia (Italian) young group; LEI, Emilia Romagna (Italian) elderly group; EEI, Emilia Romagna (Italian); YEI, Emilia Romagna (Italian) young group.
