# Supplementary material for: Gut Microbiota Composition and Metabolic Potential of Long-Living People in China
Source: Front Aging Neurosci. 2022 Jul 7;14:820108. doi: 10.3389/fnagi.2022.820108 (PMC9300991; doi:10.3389/fnagi.2022.820108)
Supplement: Supplementary file 4 [file Table_3.pdf]

Table S3. Differential taxonomic in gut microbiome with known functions between three age groups.

Genus	Species	Producing	Reference
Oscillospira		butyrate	(Gophna et al., 2017)
Odoribacter		succinate, propionate, acetate, butyrate, isobutyrate, and isovalerate	(Gomez-Arango et al., 2016)
Odoribacter		butyrate	(Morgan et al., 2012)
Anaerofustis		acetic and butyric acids	(Lawson, 2015)
Turicibacter		lactate	(Bosshard, 2015)
	Roseburia sp. 40_7	butyrate	(Duncan et al., 2002)
	Lachnospiraceae bacterium 3_1_57FAA_CT1	butyrate	(Vital et al., 2015)
	Clostridium leptum	butyrate	(Lay et al., 2005)
	Clostridium asparagiforme	acetate, lactate, and ethanol bacterium	(Mohan et al., 2006)
	Eubacterium ramulus	butyrate and acetate	(Schneider et al., 1999)
	Anaerofustis stercorihominis	acetic and butyrate	(Finegold et al., 2004)
	Clostridium celatum	acetic	(Hauschild and Holdeman, 1974)
	Porphyromonas bennonis	acetic	(Summanen et al., 2009)
	Odoribacter splanchnicus	Acetate, propionate and butyrate	(Goker et al., 2011)
	Clostridium methylpentosum	acetate and propionate	(Himelbloom and Canale-Parola, 1989)

Pyramidobacter piscolens	acetate and propionate	(Downes et al., 2009)
Peptoniphilus lacrimalis	butyrate	(Ezaki et al., 2001)
Roseburia inulinivorans	butyrate	(Scott et al., 2006)
Lactobacillus vaginalis	D/L-lactic acid.	(Embley et al., 1989)

References

Bosshard, P.P. (2015). "Turicibacter," in *Bergey's Manual of Systematics of Archaea and Bacteria*.), 1-2.

Downes, J., Vartoukian, S.R., Dewhirst, F.E., Izard, J., Chen, T., Yu, W.H., et al. (2009). Pyramidobacter piscolens gen. nov., sp. nov., a member of the phylum 'Synergistetes' isolated from the human oral cavity. *Int J Syst Evol Microbiol* 59**,** 972-980. doi: 10.1099/ijs.0.000364-0

Duncan, S.H., Hold, G.L., Barcenilla, A., Stewart, C.S., and Flint, H.J. (2002). Roseburia intestinalis sp. nov., a novel saccharolytic, butyrate-producing bacterium from human faeces. *Int J Syst Evol Microbiol* 52**,** 1615-1620. doi: 10.1099/00207713-52-5-1615

Embley, T.M., Faquir, N., Bossart, W., and Collins, M.D. (1989). Lactobacillus vaginalis sp. nov. from the Human Vagina. *International Journal of Systematic Bacteriology* 39**,** 368-370. doi: 10.1099/00207713-39-3-368

Ezaki, T., Kawamura, Y., Li, N., Li, Z.Y., Zhao, L., and Shu, S. (2001). Proposal of the genera Anaerococcus gen. nov., Peptoniphilus gen. nov. and Gallicola gen. nov. for members of the genus Peptostreptococcus. *Int J Syst Evol Microbiol* 51**,** 1521-1528. doi: 10.1099/00207713-51-4-1521

Finegold, S.M., Lawson, P.A., Vaisanen, M.L., Molitoris, D.R., Song, Y., Liu, C., et al. (2004). Anaerofustis stercorihominis gen. nov., sp. nov., from human feces. *Anaerobe* 10**,** 41-45. doi: 10.1016/j.anaerobe.2003.10.002

Goker, M., Gronow, S., Zeytun, A., Nolan, M., Lucas, S., Lapidus, A., et al. (2011). Complete genome sequence of Odoribacter splanchnicus type strain (1651/6). *Stand Genomic Sci* 4**,** 200-209. doi: 10.4056/sigs.1714269

Gomez-Arango, L.F., Barrett, H.L., Mcintyre, H.D., Callaway, L.K., Morrison, M., Dekker Nitert, M., et al. (2016). Increased Systolic and Diastolic Blood Pressure Is Associated With Altered Gut Microbiota Composition and Butyrate Production in Early Pregnancy. *Hypertension* 68**,** 974-981. doi: 10.1161/HYPERTENSIONAHA.116.07910

Gophna, U., Konikoff, T., and Nielsen, H.B. (2017). Oscillospira and related bacteria - From metagenomic species to metabolic features. *Environ Microbiol* 19**,** 835-841. doi: 10.1111/1462-2920.13658

Hauschild, A.H.W., and Holdeman, L.V. (1974). Clostridium celatum sp.nov., Isolated from Normal Human Feces. *International Journal of Systematic Bacteriology* 24**,** 478-481. doi: 10.1099/00207713-24-4-478

Himelbloom, B.H., and Canale-Parola, E. (1989). Clostridium methylpentosum sp. nov.:

a ring-shaped intestinal bacterium that ferments only methylpentoses and pentoses. *Arch Microbiol* 151, 287-293. doi: 10.1007/BF00406553

Lawson, P.A. (2015). "Anaerofustis," in *Bergey's Manual of Systematics of Archaea and Bacteria*.), 1-4.

Lay, C., Sutren, M., Rochet, V., Saunier, K., Dore, J., and Rigottier-Gois, L. (2005). Design and validation of 16S rRNA probes to enumerate members of the Clostridium leptum subgroup in human faecal microbiota. *Environ Microbiol* 7, 933-946. doi: 10.1111/j.1462-2920.2005.00763.x

Mohan, R., Namsolleck, P., Lawson, P.A., Osterhoff, M., Collins, M.D., Alpert, C.A., et al. (2006). Clostridium asparagiforme sp. nov., isolated from a human faecal sample. *Syst Appl Microbiol* 29, 292-299. doi: 10.1016/j.syapm.2005.11.001

Morgan, X.C., Tickle, T.L., Sokol, H., Gevers, D., Devaney, K.L., Ward, D.V., et al. (2012). Dysfunction of the intestinal microbiome in inflammatory bowel disease and treatment. *Genome Biol* 13, R79. doi: 10.1186/gb-2012-13-9-r79

Schneider, H., Schwiertz, A., Collins, M.D., and Blaut, M. (1999). Anaerobic transformation of quercetin-3-glucoside by bacteria from the human intestinal tract. *Arch Microbiol* 171, 81-91. doi: 10.1007/s002030050682

Scott, K.P., Martin, J.C., Campbell, G., Mayer, C.D., and Flint, H.J. (2006). Whole-genome transcription profiling reveals genes up-regulated by growth on fucose in the human gut bacterium "Roseburia inulinivorans". *J Bacteriol* 188, 4340-4349. doi: 10.1128/JB.00137-06

Summanen, P.H., Lawson, P.A., and Finegold, S.M. (2009). Porphyromonas bennonis sp. nov., isolated from human clinical specimens. *Int J Syst Evol Microbiol* 59, 1727-1732. doi: 10.1099/ijs.0.001909-0

Vital, M., Gao, J., Rizzo, M., Harrison, T., and Tiedje, J.M. (2015). Diet is a major factor governing the fecal butyrate-producing community structure across Mammalia, Aves and Reptilia. *ISME J* 9, 832-843. doi: 10.1038/ismej.2014.179
