# Supplementary material for: Gut Microbiota Composition and Metabolic Potential of Long-Living People in China
Source: Front Aging Neurosci. 2022 Jul 7;14:820108. doi: 10.3389/fnagi.2022.820108 (PMC9300991; doi:10.3389/fnagi.2022.820108)
Supplement: Supplementary file 5 [file Table_4.pdf]

Table S4 KOs corresponding to genes in the butyrate synthesis pathway by KEGG

Gene	KOs	Gene	KOs
KamA	K01843	*mgsA*	K01734
kamD	K01844	*ptb*	K00634
KamE	K18011	*eutD*	K04020
Kdd	K18012	*ackA*	K00925
Kce	K18013	*tdcD*	K00932
Kal	K18014	*pta*	K00625
L2Hgdh	K00109	*buk*	K00929
Gct	K01039、K01040	*fadM*	K00318
HgCoAd		*tesB*	K10805
Gcd	K00117	*tesA*	K10804
AbfH	K18120	*entH*	
4Hbt	K07107	*ybgC*	K07107
AbfD	K14534	*ybhC*	K01051
Isom		*yciA*	K10806
